# Supplementary material for: Antithrombotic Effects of Fostamatinib in Combination with Conventional Antiplatelet Drugs
Source: Int J Mol Sci. 2022 Jun 23;23(13):6982. doi: 10.3390/ijms23136982 (PMC9266367; doi:10.3390/ijms23136982)
Supplement: Supplementary file 1 [file ijms-23-06982-s001.zip › ijms-1755810-supplementary.pdf]

Supplementary

Reagents

Horm collagen was obtained from Takeda (High Wycombe, United Kingdom). Fostamatinib (R788) and R406 were provided by Rigel Pharmaceuticals. Rhodocytin was isolated according to a published protocol and provided by Johannes Eble from the University of Münster.[1] CRP was purchased from CambCol Laboratories (Cambridge, UK). Aspirin and ticagrelor and 3,3'-dihexyloxocarbocyanine iodide ($DiOC_6$) were obtained from Thermo Fisher Scientific (Gloucester, UK). Human fibrinogen was from Enzyme Research Laboratories (Swansea, United Kingdom).

TRAP, ADP and ASPI were purchased from Roche (Roche Diagnostics, Burgess Hill, United Kingdom). HRP-conjugated secondary antibodies and ECL autoradiography film were from Amersham Biosciences (GE Healthcare, Bucks, United Kingdom). ECL reagent was from Thermofisher (Waltham, MA). For mouse studies fostamatinib was formulated into OpenStandard diet.

Antibodies: PLCγ2 pY1217 and Syk pY525/526 were from Cell Signalling Technology (Danvers, Massachusetts, United States). Syk (4D10) and PLCγ2 (SC 407) were purchased from Santa Cruz (Wembley, UK). LAT pY200 mAb was obtained from Abcam (Cambridge, United Kingdom). Mouse anti-human anti-phospho-tyrosine (clone 4G10) mAb, LAT and α-Tubulin were purchased from Millipore UK Ltd (Watford, United Kingdom). Alexa Fluor 488 phalloidin were purchased from Invitrogen (Invitrogen, ThermoFisher Scientific).

Methods

Atherosclerotic plaque homogenate preparation

Atherosclerotic plaque samples were collected from patients with coronary artery diseases who underwent surgery for high-grade carotid artery stenosis and stored at -80°C after sampling. Cryosections were taken from each atherosclerotic plaque specimen for histological analysis purposes. Then all atherosclerotic plaque specimens were placed in one 500 ml borosilicate glass laboratory reagent bottle immersed in liquid nitrogen (-196°C) and manually crushed with glass mortar until the fine powder was obtained. 20 ml of PBS was then added to the glass containing atherosclerotic plaque homogenate, thawed at 37 °C for 3 min, transferred into 50 ml falcon tubes. Atherosclerotic plaque homogenate was sonicated (10 times for 5 seconds at 12% amplitude) on ice for tissue dispersal, followed by centrifugation (1200 rpm for 1 min). The supernatant was retrieved, and protein concentration measured with a standard Bicinchoninic Acid (BCA) protein assay (Thermo Fisher Scientific, Gloucester, UK).

Preparation of platelet-rich plasma (PRP)

Blood tubes are centrifuged at 200 x *g* for 20 minutes. After centrifugation, the platelet-rich plasma (PRP) is collected and the remaining blood is centrifuged at 1000 x *g* for 10 minutes to collect platelet-poor plasma (PPP) to be used as a blank for LTA aggregometer.

Preparation of washed platelets (WP)

Washed platelets were prepared by centrifugation of citrated blood with acid/citrate/dextrose (ACD) at 9:1 v:v at 200 x *g* for 20 min. PRP was obtained and centrifuged at 1000 x *g* for 10 min with the presence of 2.8 µM prostacyclin (Cayman Chemicals). The supernatant was discarded, and the platelet pellet was resuspended

in 2 ml modified Tyrode's buffer (134 mM NaCl, 0.34 mM Na_2HPO_4, 2.9 mM KCl, 12 mM $NaHCO_3$, 20 mM HEPES, 5 mM glucose, 1 mM $MgCl_2$; pH 7.3) and adding ACD and 2.8 μM prostacyclin before being centrifuged for 10 min at 1000 x *g*. The platelet pellet was resuspended in modified Tyrode's buffer. The platelet count was adjusted a Coulter Counter (Beckman Coulter) to 2 $x10^8$/ml for platelet aggregation testing, 4-5 $x10^8$/ml for immunoblotting or 2 $x10^7$/ml for spreading. Washed platelets were left for 30 min before platelet function and aggregation tests to ensure complete PGI_2 degradation.

Light transmission aggregometry (LTA)

Platelet aggregation studies were performed using a PAP-E8 aggregometer (Bio/Data Corp, Horsham, PA). Platelets (PRP or washed platelets [WP]) were preincubated with different concentrations of inhibitors at 37 °C for 10 min in the incubation wells. The final concentration of aspirin and ticagrelor added ex vivo throughout this study were 30 μM and 1 μM, respectively. The tubes were then transferred into stirred incubation wells and incubated under stirring conditions for 2 min. Tubes were then moved to test wells, and aggregation was stimulated by adding an agonist. Multiple runs of different concentrations of the inhibitor with different concentrations of agonist were carried out to assess the inhibitory effect of the inhibitor on platelet aggregation induced by an agonist.

Multiplate impedance aggregometry (MEA)

Whole blood aggregation was determined using multiple electrode aggregometry (Multiplate® Analyzer, Roche Diagnostics, Mannheim, Germany). 300 μl of blood was preincubated in the test cells (Multiplate® mini test cells, Roche Diagnostics, Mannheim, Germany) for 10 min at 37 °C with different concentrations of the inhibitors. The samples were stirred using a disposable PTFE (Poly-tetra-fluoro-ethylene) -

coated magnetic stirrer. After three min of incubation, preheated (37°C) normal saline (300µl) is added to the sample. At the end of the 10 min incubation, platelets are stimulated with an agonist. Multiple runs of different concentrations of the inhibitor with different concentrations of agonist were carried out to assess the inhibitory effect of the inhibitor on platelet aggregation induced by an agonist.

Optimul assay

Optimul assay 96 well plates were prepared as previously described.[2] Wells were filled with different concentrations of lyophilised platelet agonists with the following concentrations; Arachidonic Acid (AA) (0.03-1 mM; Sigma-Aldrich, Poole, UK), ADP (0.005-40 mM; Sigma- Aldrich, Poole, UK), epinephrine (0.0004-10 mM; Labmedics, Stockport, UK), collagen (0.01-40 mg/mL; Nycomed, Linz, Austria), TRAP-6 amide (SFLLRN; 0.03-40 mM, Bachem, St. Helens, UK), U46619 (0.005-40 mM; Labmedics, Stockport, UK), and ristocetin (0.14-4 mg/mL; Helena Biosciences, Tyne and Wear, UK). PRP or PPP (40 mL) was added to the appropriate agonist-free control. The plate was placed on a heater/shaker (BioShake iQ; Q Instruments, Jena, Germany) at 37 °C to mix at 1200 rpm for 5 minutes. Absorbance was then measured at 595 nm on a 96-well plate reader (VersaMax Microplate reader; Associates of Cape Cod Inc., East Falmouth,MA). Platelet aggregation was expressed as the maximal percent change in light transmittance from PRP wells in response to agonists, using PPP wells as reference.

Protein phosphorylation

Washed platelets were pre-treated with 9 µM eptifibatide to block αIIbβ3-mediated aggregation. Stimulation with an agonist was done under stirring (1200 rpm) at 37 ºC for 180 sec. Stimulation was performed in the presence of either vehicle (DMSO) or R406 (0.1 – 10 µM). The reaction was stopped by the addition of 5x SDS reducing

sample buffer (100 µl). The lysates were incubated with the appropriate antibody and run on an SDS-PAGE, transferred and western blotted. Membranes were imaged using ECL (Fisher Scientific) and analysis was done with image studio lite for band intensity measurement.

Platelet adhesion and thrombus formation under flow conditions

Ibidi u-Slide VI 0.1 Uncoated chamber (ibidi®, Martinsried, Germany) were coated with 200µg/ml Horm collagen or Ibidi µ-Slide VI 0.1 ibitreat chamber were coated with 1 mg/ml plaque overnight and kept at 4°C. Then 1 hour before carrying out flow adhesion experiment chamber blocked with 4 mg/ml BSA made in PBS and kept again at 4°C. Flow studies were done using citrated whole blood from healthy or patient blood. Blood was incubated for 10 min at 37 ºC with 2 µM $DiOC_6$ for platelet visualisation along with DMSO vehicle or inhibitors. Blood was then perfused at shear rate 1000 s^{-1} over immobilised collagen or plaque for 10 min using PHD 2000 Syringe Pump (Harvard Apparatus, Holliston, Massachusetts, USA). Platelet adhesion and Thrombus formation were continuously captured by recording z-stacks (41 planes/step size 0.5 µm) every 30 seconds for two separate locations by an Evos ® FL Auto imaging system (Thermo Fisher Scientific, Gloucester, UK) using a 20x objective for the entire flow period. Z-stacks for each condition were then analysed using image J (version 1.52, NIH, USA) with an in-house macro that generates platelet intensity and surface coverage of thrombi for each time point.

Platelet spreading

Round glass coverslips were coated with collagen, plaque, fibrinogen or fibrin and placed in a 24 well plate for 1 hour at RT. After 1 hour, coverslips were washed once with PBS and blocked with BSA (5 mg/mL) in PBS for 60 mins at RT. In the fibrin wells, thrombin was neutralised by adding PPACK. Washed human platelets ($2x10^7$ ml^{-1}

prepared in Modified Tyrode's buffer) were preincubated with DMSO vehicle or inhibitors at 37°C for 30 mins. Platelets were then allowed to spread on the coated coverslips for 30 min at 37 ºC. Following spreading, coverslips were washed with PBS once to remove nonadherent platelets and then fixed with 4% neutralised formalin for 10 min. Following three times PBS washing, platelets were permeabilised with 0.1 % (v/v) Triton X-100 for 5 min and then washed with PBS to be labelled with actin stain phalloidin-Alexa488 (Thermo Fisher Scientific, Gloucester, UK). Platelets were washed and mounted with Hydromount (National Diagnostics, Hull, UK) for epifluorescence imaging. Images were acquired using an Axio Observer 7 inverted epifluorescence microscope (Carl Zeiss AG, Oberkocken, Germany) using 64x 1.4 NA oil immersion objective lens. Five images were acquired for each condition and were analysed using a validated analysis method.[3]

Flow cytometry

Flow cytometry was performed on a BD Accuri C6 flow cytometer, and results were analysed using BD Accuri C6 software. Platelet activation following stimulation with indicated agonists was determined using anti-P-selectin FITC and anti-activated integrin αIIβ3 (JON/A) PE antibodies (Emfret Analytics Eibelstadt, Germany).

In vivo thrombosis models

Ferric chloride ($FeCl_3$)–induced injury of carotid arteries were performed in mice (20-25 g) as previously described.[4] Briefly, mice were anesthetised, carotid arteries exposed and thrombosis initiated by topical application of FeCl3-soaked filter paper (10%, 3 min). Accumulation of platelets labelled with anti-GPIbβ DyLight488 (Emfret Analytics) infused intravenously prior to injury were then captured and analysed using Slidebook6 software (Intelligent Imaging Innovations).

The inferior vena cava (IVC) stenosis (partial flow restriction) model was performed as described.[5] Briefly, mice (20-25g) were anaesthetised, laparotomy performed and bowels exteriorised. IVC side branches were then closed and the IVC was ligated over a 30-gauge spacer which was then removed resulting in 90% reduction in vessel lumen diameter. The peritoneum was then closed and mice allowed to recover. After 48 hours, mice were culled and size, weight and prevalence of IVC thrombi assessed.

Determination of R406 plasma concentration in treated mice

Plasma samples were analysed to determine the concentrations of fostamatinib and its metabolite R406 using a validated liquid-liquid extraction using methyl tert-butyl ether (HPLC Grade, Fisher Scientific), turbo ion spray LC/MS/MS assays.

Supplementary figures

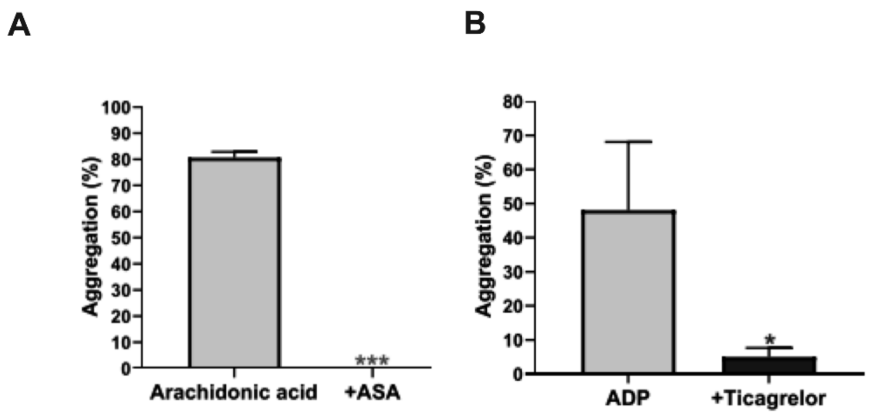

Supplementary Figure S1. The effect of chosen concentration of aspirin and ticagrelor.

A) Inhibition of Arachidonic acid (AA)-stimulated platelet aggregation by 30 µM aspirin. B) Inhibition of ADP-stimulated platelet aggregation by 1 µM ticagrelor. The effect of combinations of 30 µM aspirin and 1 µM ticagrelor was compared to stimulated vehicle control and assessed using paired t-test. (n=6, *P <0.05, **P <0.01, and ***P <0.001).

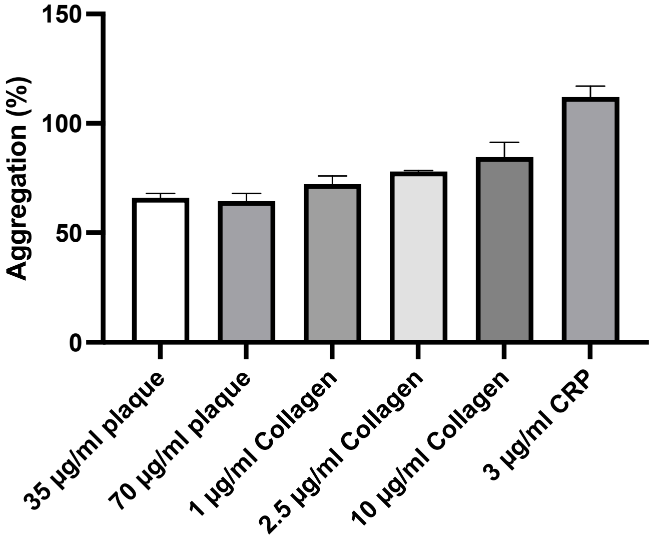

Supplemental Figure S2. Atherosclerotic plaque causes low-degree GPVI stimulation as shown by platelet aggregation response in PRP samples in LTA.

Atherosclerotic plaque homogenate stimulated platelet aggregation in PRP in LTA, inducing a weaker aggregation response than low collagen and CRP, n=3.

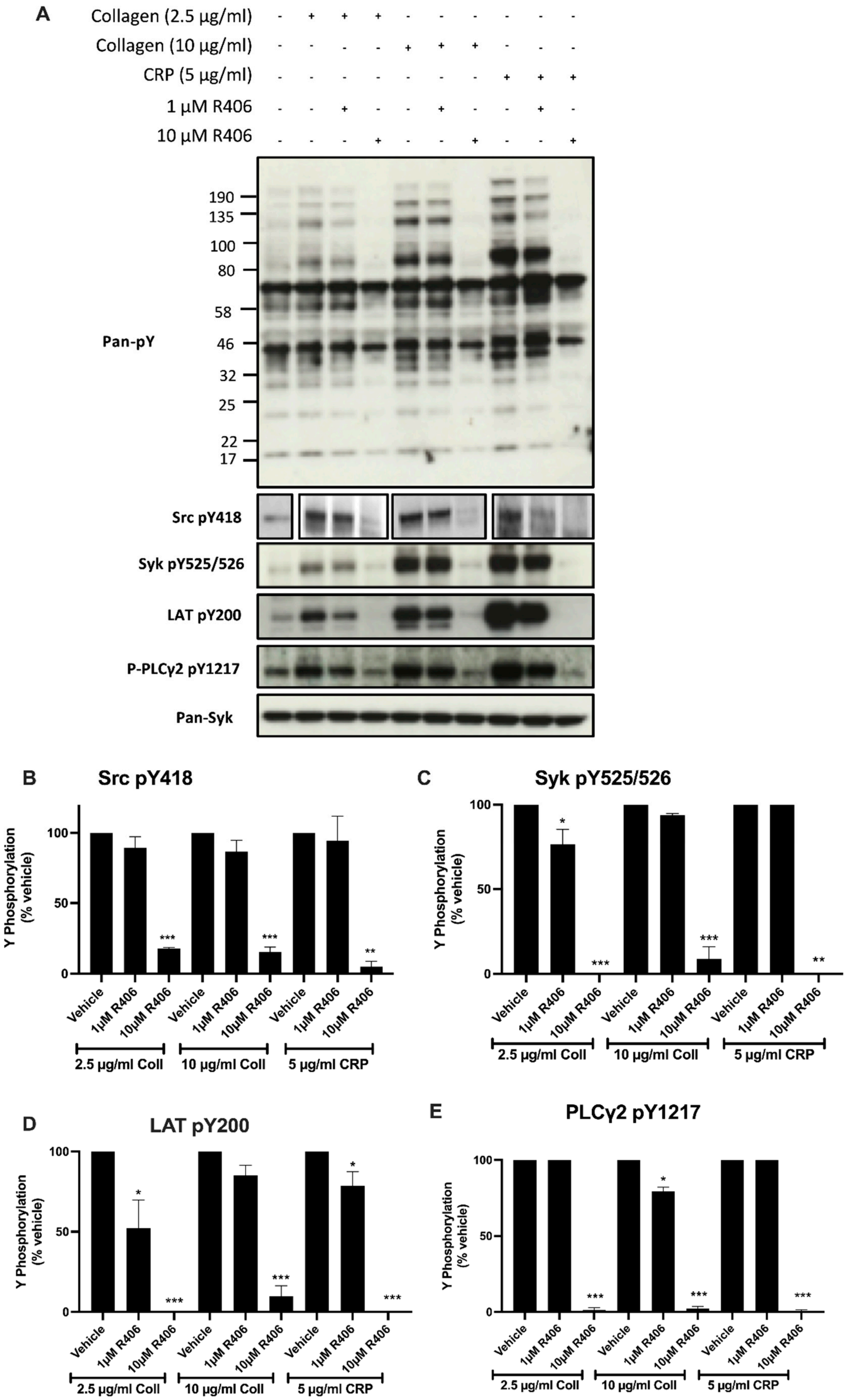
A
Collagen (2.5 µg/ml)
Collagen (10 µg/ml)
CRP (5 µg/ml)
1 µM R406
10 µM R406
Pan-pY
Src pY418
Syk pY525/526
LAT pY200
P-PLCγ2 pY1217
Pan-Syk
B
Src pY418
C
Syk pY525/526
D
LAT pY200
E
PLCγ2 pY1217
Y Phosphorylation (% vehicle)
Vehicle
1µM R406
10µM R406
2.5 µg/ml Coll
10 µg/ml Coll
5 µg/ml CRP

Supplementary Figure S3. R406 blocks Src, Syk, LAT and PLCγ2 phosphorylation following activation of GPVI.

Healthy donor washed human platelets (4 x10^8/ml) were incubated with R406 or vehicle (0.1% DMSO) for 10 minutes, then stimulated with Horm collagen or collagen-related peptide (CRP) for 3 minutes in the presence of eptifibatide (9 µM). Platelets were then lysed, separated with SDS-PAGE and Western blotted for tyrosine phosphorylation (pY). A) Representative images of 3 independent experiments. Densitometry quantification of (B) Src pY418 (C) Syk pY525/526 (D) LAT pY200 and (E) PLCγ2 pY1217 Band intensities were normalised to vehicle control. Mean ± SEM. One-way ANOVA with Dunnett's correction for multiple comparisons, * $P < 0.05$, ** $P < 0.01$, *** $P < 0.001$.

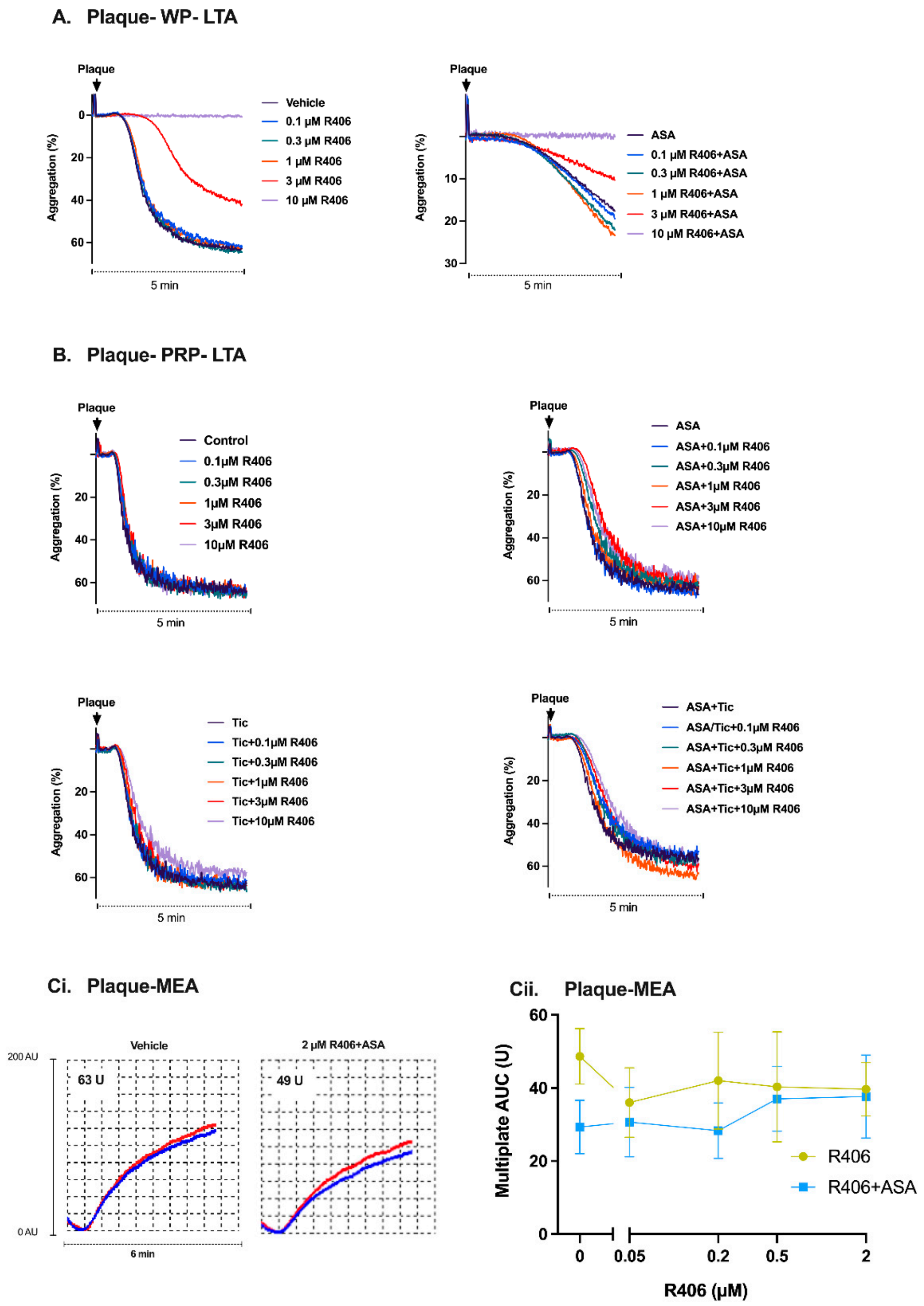

Supplementary Figure S4. Representative traces for figure 4 of the effect of R406 combined with ASA and ticagrelor on atherosclerotic plaque-induced platelet aggregation.

GPVI-mediated platelet aggregation in healthy donor PRP by A) washed platelets B) PRP assessed with LTA C) hirudin anticoagulated whole blood assessed by MEA. Samples were incubated with either vehicle (0.1 % DMSO), R406, 30 μM aspirin or 1 μM ticagrelor for 10 min, then were stimulated with 70 μg/ml atherosclerotic plaque to induce aggregation. ASA, Aspirin. Tic, Ticagrelor.

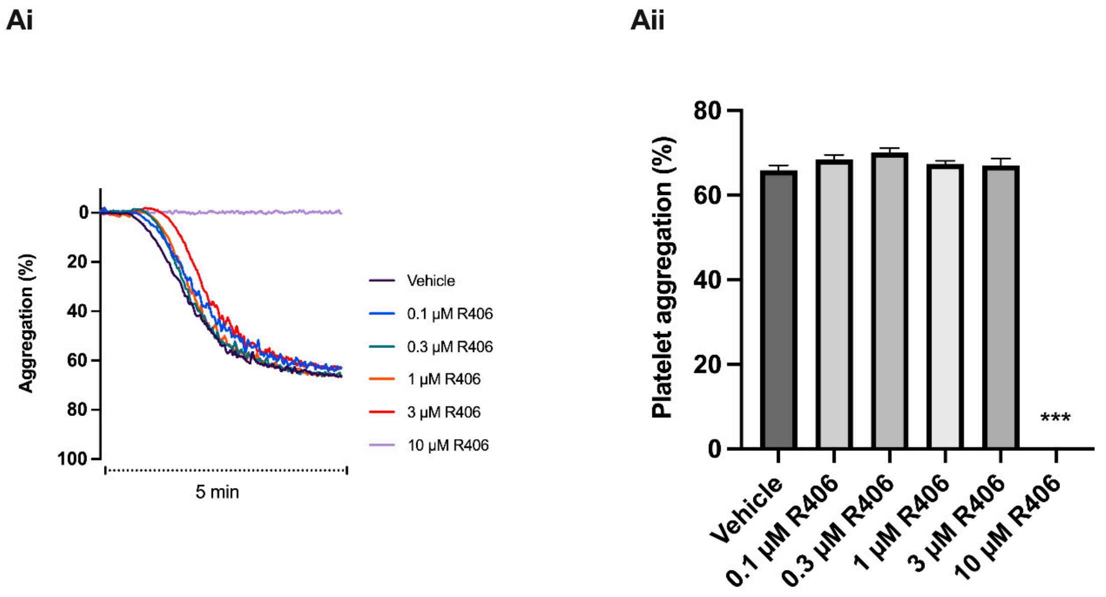

Supplementary Figure S5. The effect of R406 on platelet aggregation in washed platelets assessed with LTA.

5 µg/ml collagen-mediated platelet aggregation in healthy donor washed platelets by LTA. Samples were incubated with either vehicle (0.1 % DMSO) or R406 for 10 min, then were stimulated with 5 µg/ml collagen to induce aggregation. (Ai) Representative trace of all conditions. (Aii) Quantification of effect of different concentrations of R406 on collagen-induced platelet aggregation. Results presented as mean ± SEM and compared to vehicle control by one-way ANOVA with Dunnett correction for multiple comparisons. n=4-7, * P < 0.05, ** P < 0.01, *** P < 0.001.

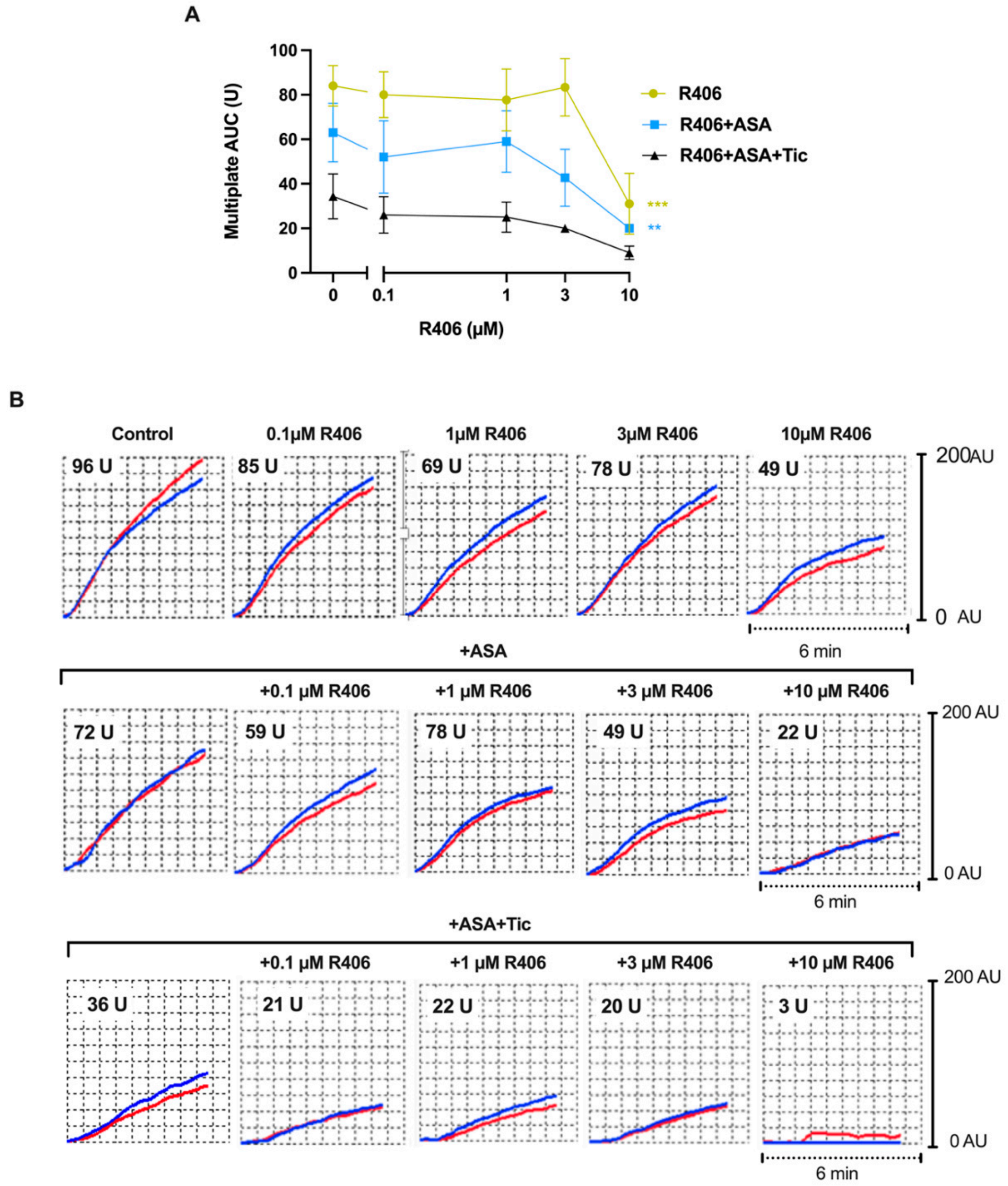

Supplementary Figure S6. Effect of R406 with aspirin and ticagrelor on collagen-induced platelet aggregation in whole blood assessed by multiple electrode aggregometry (MEA).

A) Dose-response curve of R406 alone and with aspirin and ticagrelor on 3.2 μg/ml Horm collagen-induced platelet aggregation in hirudin anticoagulated blood. Results are shown as mean ± SEM of three independent experiments. B) Representative traces from a single MEA experiment, where hirudin anticoagulated human whole blood was preincubated with different concentrations of R406, aspirin, aspirin and ticagrelor or vehicle (0.1 % DMSO) for 10 min at 37 °C and stimulated with 3.2 μg/ml Horm collagen, then aggregation was monitored for 6 min in Multiplate® analyser. Statistical analysis using two-way ANOVA with Tukey's correction for multiple comparisons compared to respective controls (Vehicle, aspirin or aspirin+ticagrelor) (* P <0.05, ** P <0.01, and *** P <0.001).

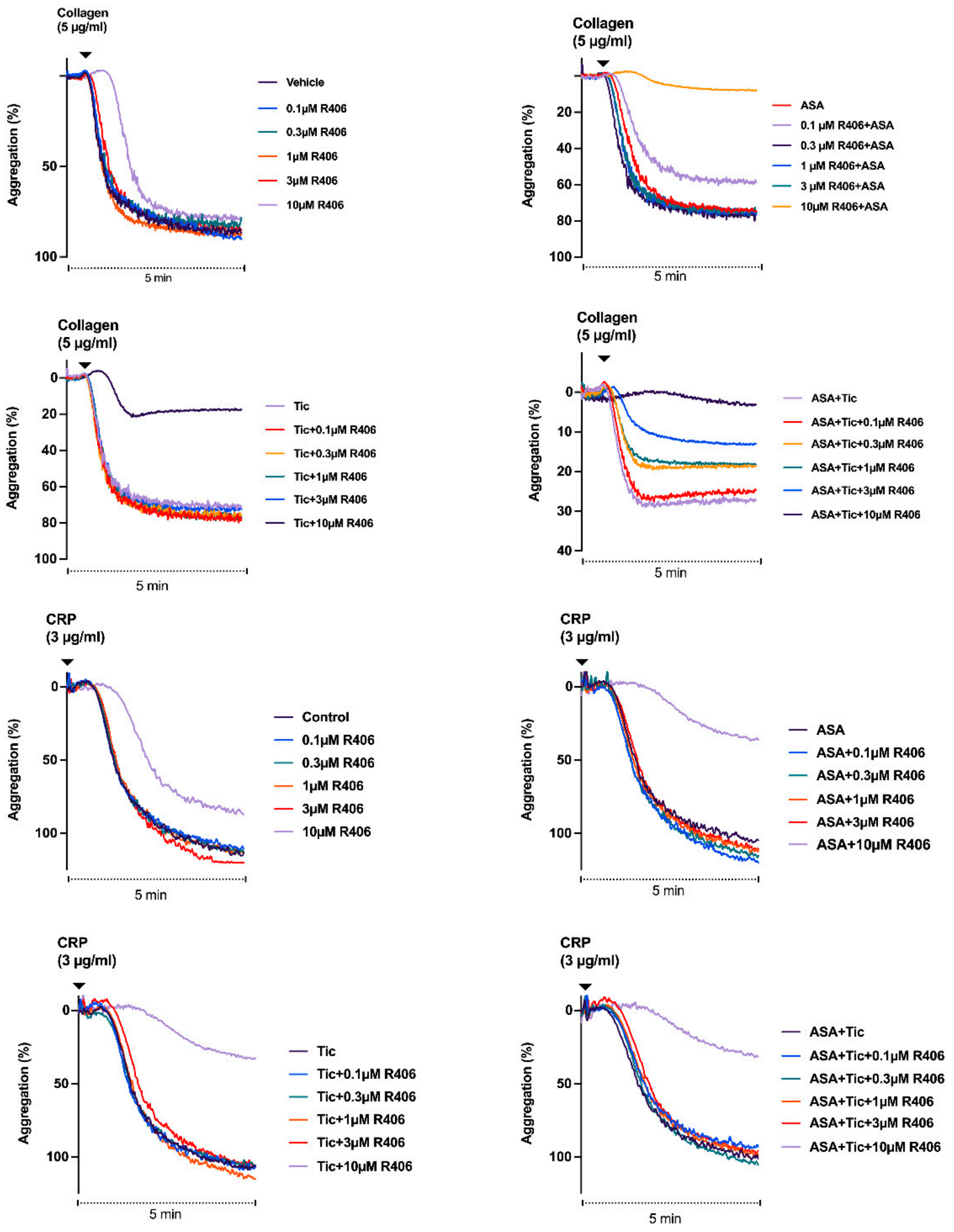

Supplementary Figure S7. Representative traces for figure 3 for the effect of R406 combined with ASA and/or ticagrelor on collagen- and CRP-induced platelet aggregation in PRP measured by LTA.

GPVI-mediated platelet aggregation in healthy donor PRP by LTA. Samples were incubated with either vehicle (0.1 % DMSO), R406, 30 μM aspirin or 1 μM ticagrelor for 10 min, then were stimulated with 5 μg/ml collagen or 3 μg/ml CRP to induce aggregation. Representative trace of all conditions. ASA, Aspirin. Tic, Ticagrelor.

Characteristic		
Age – median (IQR)		62 (56, 72.5)
Male gender – no. (%)		10/12 (83%)
Ethnicity		
	Caucasian – no. (%)	8/12 (67%)
	Black – no. (%)	1/12 (8%)
	Asian – no. (%)	3/12 (25%)
Current smoker – no. (%)		5/12 (42%)
Hypertension – no. (%)		5/12 (42%)
Diabetes mellitus – no. (%)		1/12 (8%)
Current presentation STEMI – no. (%)		7/12 (58%)
Current presentation NSTEMI – no. (%)		5/12 (42%)
Current presentation unstable angina– no. (%)		0/12 (0%)
Prior history of MI – no. (%)		2/12 (17%)
Prior history of PCI – no. (%)		1/12 (8%)
Previous coronary bypass grafting – no. (%)		1/12 (8%)
Heart failure – no. (%)		1/12 (8%)
Stroke – no. (%)		0/12 (0%)
Concomitant medications		
	Aspirin	12/12 (100%)
	Ticagrelor	12/12 (100%)
	Beta blocker	9/12 (75%)
	ACE inhibitor	8/12 (67%)
	Statin	12/12 (100%)

Supplementary Figure S8. Clinical characteristics of patients taking aspirin and ticagrelor.

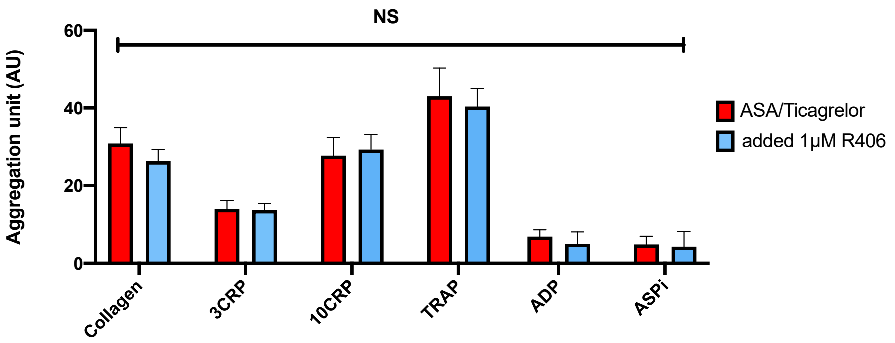

Supplementary Figure S9. R406 1 μM does not promote further platelet inhibition in patient samples on dual antiplatelet therapy (DAPT).

Aggregation results from MEA in response to: collagen 3.2 μg/ml, CRP 3 μg/ml, CRP 10 μg/ml, TRAP 32 μM, ADP 6.5 μM, ASPi 0.5 mM. Patient samples on ASA/ticagrelor incubated for 10 min under stirring conditions either with or without R406. Data are shown as mean ± SEM.

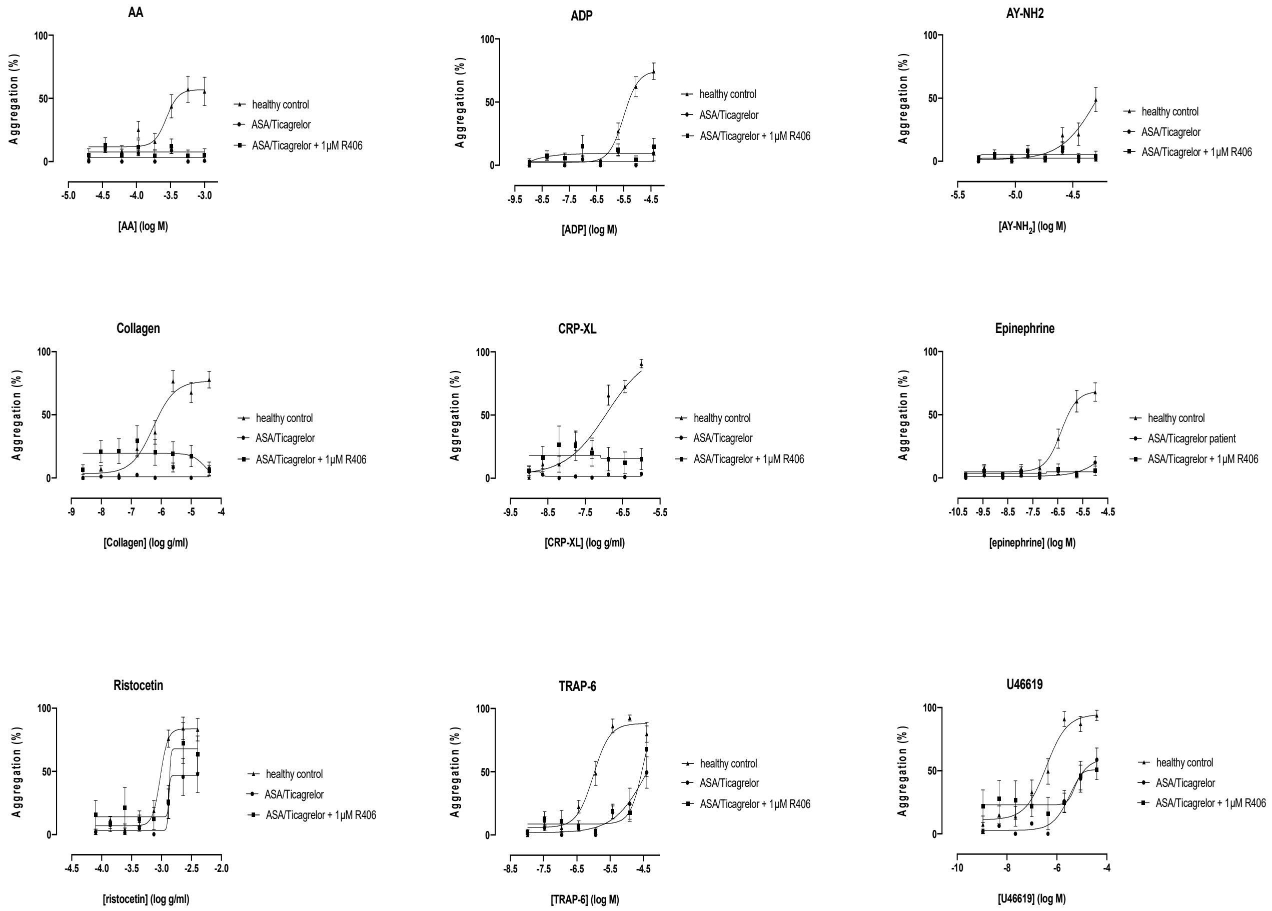

Supplementary Figure S10. 1 µM R406 does not promote further platelet inhibition in patient samples on dual antiplatelet therapy (DAPT).

Dose-response curves from Optimul assay in response to: AA (0.03–1 mM), ADP (0.005–40 µM), AY-NH2 (6.6–50 µM), collagen (0.001–40 µg/ml), CRP (0.0023–1 µg/ml), epinephrine (0.0004–10 µM), ristocetin (0.14–4 mg/ml), TRAP-6 amide (0.03–40 µM) and U46619 (0.005–40 µM) on 96-well plates after 5 minutes of mixing (1200 rpm) at 37°C or 5 min of incubation with R406. n=15 healthy volunteers and n=8 patient samples. Data are shown as mean ± SD.

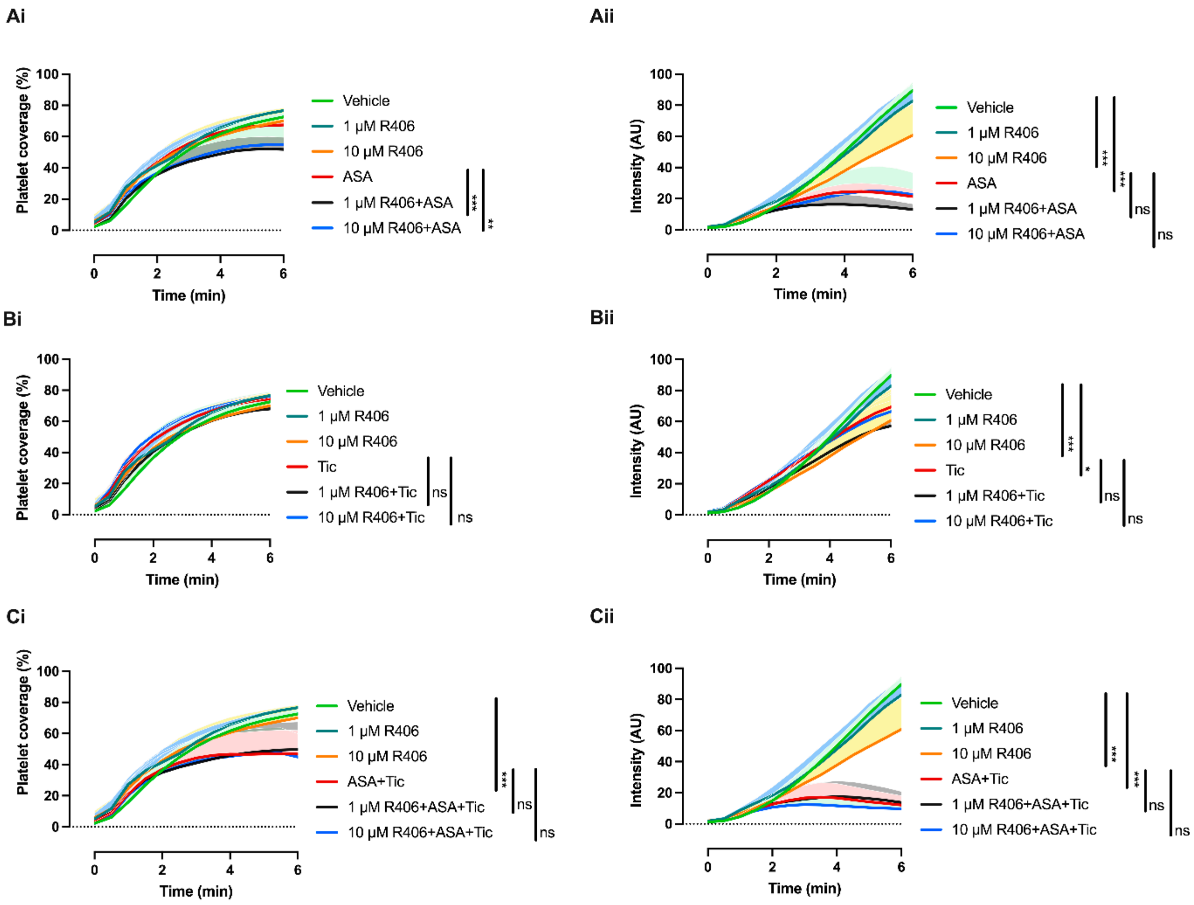

Supplementary Figure S11. R406 inhibits platelet aggregate formation (intensity) but not platelet adhesion over collagen.

Healthy donor whole blood was incubated for 10 min at 37°C with R406, 30 µM aspirin and/or 1 µM ticagrelor then perfused over 200 µg/ml collagen coated chambers at a shear rate of 1000 s^{-1}. For visualisation, platelets were labelled with 2 µM $DiOC_6$ for 10 min prior to perfusion. (Ai, Bi, Ci) Quantification of platelet adhesion (platelet coverage) and (Aii, Bii, Cii) platelet aggregate size (fluorescence intensity) of (A) R406±aspirin (B) R406 ± ticagrelor and (C) R406 ± aspirin + ticagrelor. Measurements were taken every 30 sec. Mean (solid line) + SEM (shaded area); n=3. Statistical comparisons were made at 6 min vs vehicle control or vs aspirin and/or ticagrelor using two-way ANOVA with Dunnett's correction for multiple comparisons. * P <0.05, ** P < 0.01, *** P < 0.001. ASA, aspirin. Tic, ticagrelor.

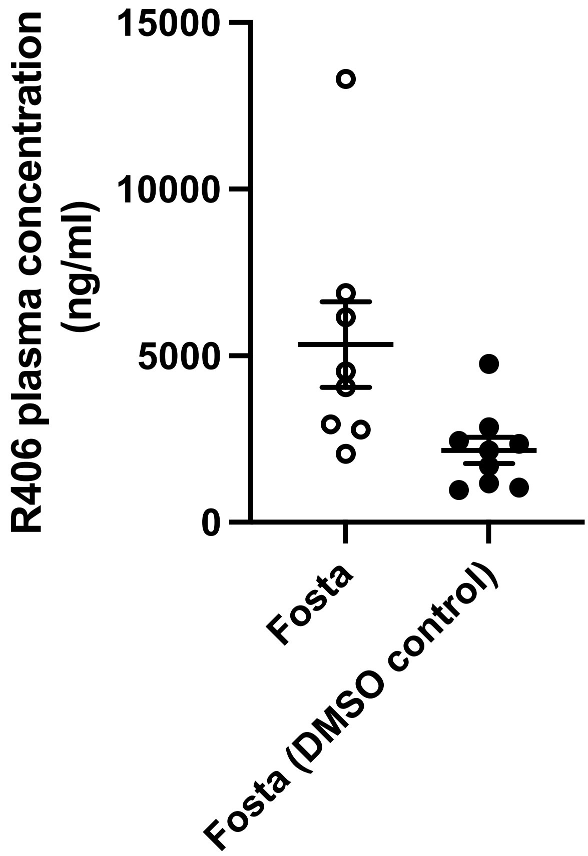

Supplemental Figure S12. R406 plasma concentrations in fostamatinib treated mice.

Concentrations of R406 active metabolite were measured in mouse plasma following oral gavage of mice with 80 mg/kg fostamatinib in vehicle or in vehicle plus DMSO (2%). Plasma samples were collected 1-hour post oral dosing and R406 metabolite measured as previous.[6] DMSO addition was required for fostamatinib combination treatment with aspirin and ticagrelor. Data shown as mean ± SEM. N = 8 – 9 mice per treatment.

References:

1. Suzuki-Inoue, K.; Fuller, G.L.J.; García, Á.; Eble, J.A.; Pöhlmann, S.; Inoue, O.; Gartner, T.K.; Hughan, S.C.; Pearce, A.C.; Laing, G.D.; et al. A novel Syk-dependent mechanism of platelet activation by the C-type lectin receptor CLEC-2. *Blood* **2006**, *107*, 542–549. https://doi.org/10.1182/blood-2005-05-1994.
2. Chan, M.V.; Warner, T.D. Standardised optical multichannel (optimul) platelet aggregometry using high-speed shaking and fixed time point readings. *Platelets* **2011**, *23*, 404–408. https://doi.org/10.3109/09537104.2011.603066.
3. Pike, J.A.; Simms, V.A.; Smith, C.W.; Morgan, N.V.; Khan, A.O.; Poulter, N.S.; Styles, I.B.; Thomas, S.G. An adaptable analysis workflow for characterization of platelet spreading and morphology. *Platelets* **2020**, *32*, 54–58. https://doi.org/10.1080/09537104.2020.1748588.
4. Smith, C.W.; Thomas, S.G.; Raslan, Z.; Patel, P.; Byrne, M.; Lordkipanidzé, M.; Bem, D.; Meyaard, L.; Senis, Y.A.; Watson, S.P.; et al. Mice Lacking the Inhibitory Collagen Receptor LAIR-1 Exhibit a Mild Thrombocytosis and Hyperactive Platelets. *Arter. Thromb. Vasc. Biol.* **2017**, *37*, 823–835. https://doi.org/10.1161/atvbaha.117.309253.
5. Payne, H.; Ponomaryov, T.; Watson, S.P.; Brill, A. Mice with a deficiency in CLEC-2 are protected against deep vein thrombosis. *Blood* **2017**, *129*, 2013–2020. https://doi.org/10.1182/blood-2016-09-742999.
6. Baluom, M.; Grossbard, E.B.; Mant, T.; Lau, D.T.W. Pharmacokinetics of fostamatinib, a spleen tyrosine kinase (SYK) inhibitor, in healthy human subjects following single and multiple oral dosing in three phase I studies. *Br. J. Clin. Pharmacol.* **2013**, *76*, 78–88. https://doi.org/10.1111/bcp.12048.
